# Desmoplastic infantile astrocytoma/ ganglioglioma in a pediatric onset multiple sclerosis patient: A case report

**DOI:** 10.1002/ccr3.9290

**Published:** 2024-08-07

**Authors:** Shima Jahani, Abdorreza Naser Moghadasi

**Affiliations:** ^1^ Multiple Sclerosis Research Center, Neuroscience Institute Tehran University of Medical Sciences Tehran Iran

**Keywords:** benign tumor, desmoplastic infantile astrocytoma, desmoplastic infantile ganglioglioma, multiple sclerosis, pediatric multiple sclerosis

## Abstract

Here we present a co‐occurrence of a non‐typical presentation of DIG/DIA and multiple sclerosis in a 13‐year‐old female. Our case highlights how a thorough investigation prior to treatment is needed in patients with such condition to choose proper management for better prognosis.

## 1 INTRODUCTION

According to the 2016 World Health Organization tumor classification, Desmoplastic infantile astrocytoma (DIA) and Desmoplastic infantile ganglioglioma (DIG), are classified as one entity among benign “neuronal and mixed neuronal‐glial” category.^1^, ^2^ These rare pediatric neuroepithelial tumors are characterized as dura based astrocytoma's with a desmoplastic reaction. This condition was first described by Taratuto,^3^ who reported six cases of “superficial cerebral astrocytoma attached to dura”, these tumors were further investigated by Vandenberg^4^ and colleagues histopathology.

Although there have been rare non infantile cases of this condition majority of affected patients are less than 2 years old. Tumor location varies as it can stick to different sites specifically periphery of the supratentorial compartment. This tumor has a heterogenous histopathological presentation, though it is mostly presented as a large mass with nodular enhancement pattern and typical undifferentiated components. This display suggest probable aggressive malignancy, thought in case of these tumors prognosis has been great. DIA/DIG management is complicated by their tumor huge size and affected patients age.^5^ There have been rare malignant cases of this condition which have led to mortality, specifically in post resection recurrent cases.^6^, ^7^

Multiple sclerosis is a neuroinflammatory disease that mostly affects young adults and in less common cases, children. Only 3%–10% of MS patients, experience their first symptom when they are less than 16 years old.^8^ Even though coexistence of central nervous system neoplasms with MS is a very rare occurrence, there have been prior reports of this condition in literature. For instance following the first case in 1912, there have been reports of this association specifically with glioblastoma and oligodendroglioma tumors.^9^, ^10^, ^11^

To our best knowledge there are no reports of co‐occurrence of the non‐infantile DIA/DIG with pediatric MS. Here we describe a 13‐year‐old girl with such characteristics.

## 2 CASE PRESENTATION

### 2.1 Case history

A 13‐year‐old female with no known past medical history visited neurology clinic complaining from paresthesia in right arm and chest area. Both systemic and neurologic examination revealed no abnormalities and patient was not taking any medication prior to her appointment.

### 2.2 Investigations, diagnosis, and treatment

**FIGURE 1:**
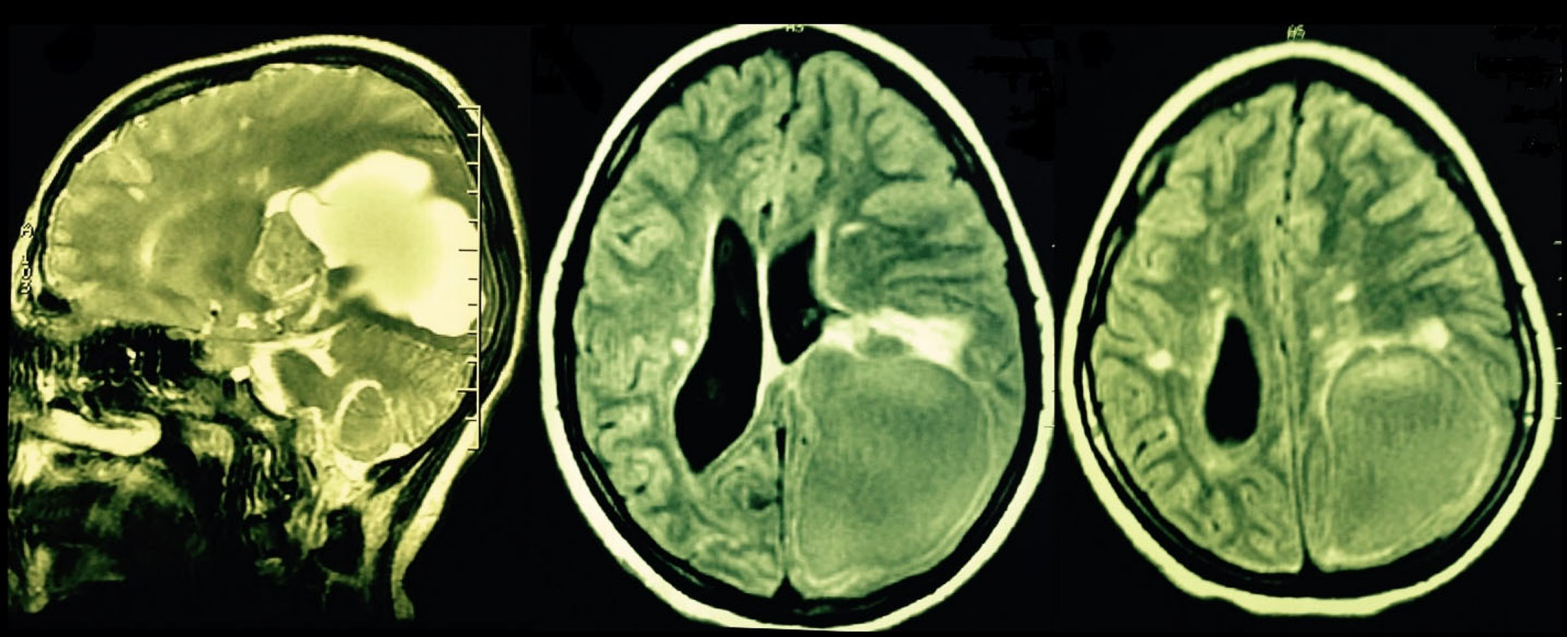
Brain MRI revealed large homogenous brain lesion in left occipital lobe in favor of brain tumor. In addition, it showed periventricular and juxtacortical hyperintense lesions in both hemispheres.

**FIGURE 2:**
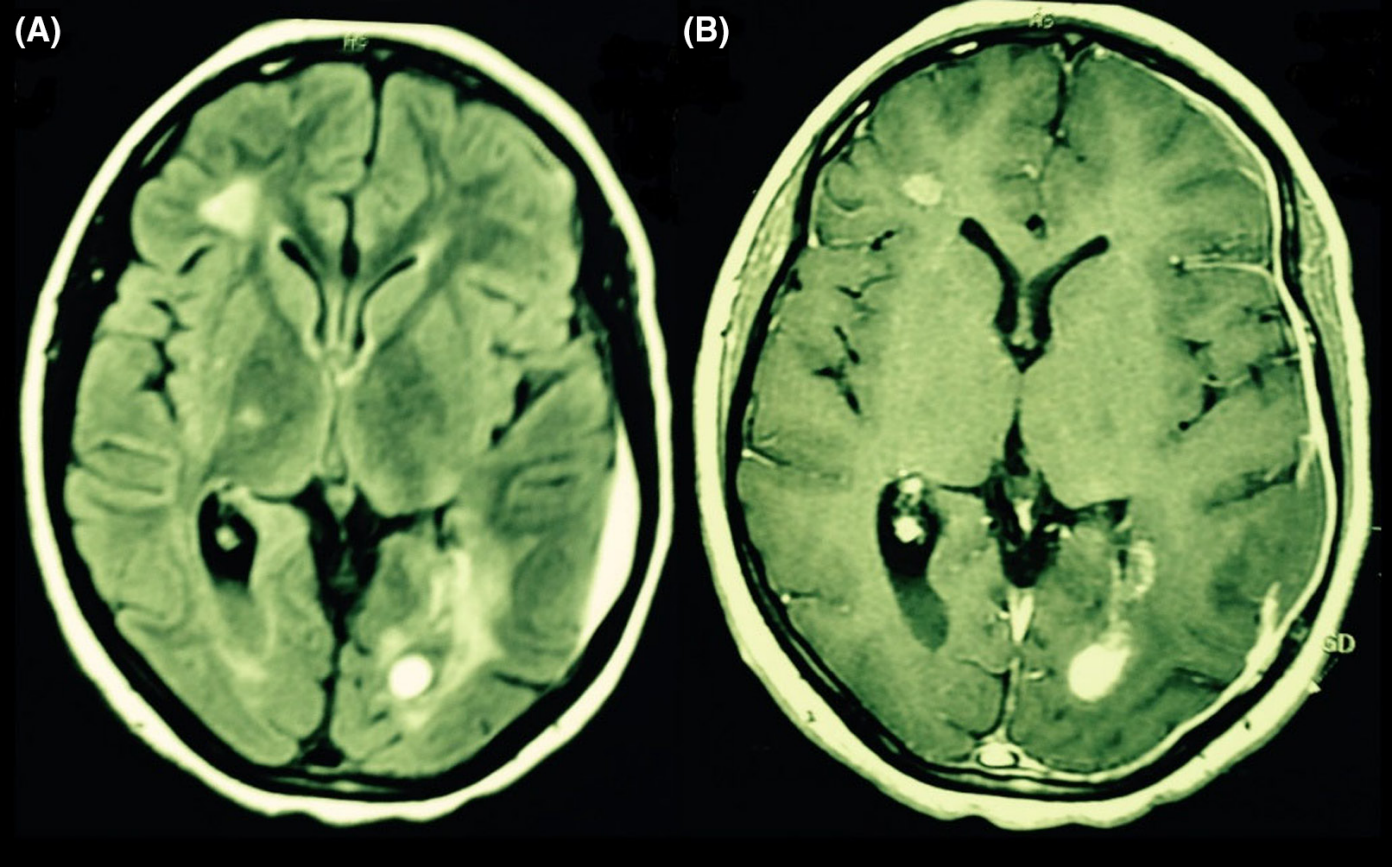
(A) Periventricular and subcortical lesions were revealed in brain MRI and some of them were new. (B) Enhancement was seen in at least three lesions after gadolinium injection.

Initial brain and spinal Magnetic resonance imaging (MRI), showed a large brain tumor in left occipital lobe with a mass effect, also periventricular and juxtacortical demyelinating lesions were detected (Figure 1), she was then scheduled for a total tumor resection surgery. Biopsy was taken during the operation which resulted in identifying tumor as DIA/DIG. Two months after surgery she experienced blurred vision in right eye and pain on motion. Ophthalmologic examination was normal and optic neuritis diagnosis was confirmed. Another brain MRI was requested which showed enhancing periventricular demyelinating plaques (Figure 2). Based on her clinical presentation and imaging results, the diagnosis of MS related optic neuritis was made and she was started on corticosteroid pulse therapy for 5 days.

### 2.3 Outcome

After treatment she showed complete visual recovery.

## 3 DISCUSSION

To summaries we presented a rare case of co‐occurrence of DIA/DIG and MS in a pediatric patient, a unique diagnostic and treatment challenge. DIA/DIG are a group of infancy tumor mostly diagnosed in 5–6‐month‐old infants. Males are more susceptible to this condition within the first 24 month of their life. DIA/DIG is known to be linked with BRAF gene mutation, which is a mutation associated with various other neoplasms such as melanoma and glioma. Initial presentation often include psychomotor delay, macrocephaly and neuroencephalopathy with a predilection for the frontal and parietal lobes.^6^, ^12^, ^13^ Also there have been reports of tumors cerebrospinal metastasis.^14^

Imaging findings commonly reveal a large mass with heterogenic components consisting of a cystic section deeper within the tumor and a superficial solid part surrounded with edema. Solid section has a hypointense presentation both in T1 and T2 cuts while Cystic component hyperintense on T2 sequences.^13^

Even though this tumor is known to have excellent prognosis after total resection surgery, risk of recurrence in rare cases should be considered. There have been reports of malignant transformation of DIA/DIG mostly to glioblastoma multiforme during 4.2 ± 3.6 years from diagnosis. Total resection and absence of leptomeningeal metastasis at presentation significantly improved affected patient's survival rate. It is widely believed that vascular disturbance following resection operation affects the residual DIA/DIGs and lead to their regression. Occasionally adjuvant therapy such as chemotherapy or radiotherapy is added to treatment, to completely suppress the residual tumor. Reports from a prior cohort shows total or partial resection is the most common treatment performed for more than 90% of patients. Seventeen percent of this cohort patients received chemotherapy and 9.6 patients underwent radiotherapy. In our case probably adjuvant chemotherapy could have prevented the MS attack.^15^

Our case is an uncommon simultaneous occurrence of two rare neurological disorders. To explain this phenomenon, we present two hypotheses about their probable shared pathophysiological mechanisms or susceptibility factors: first it is possible that genetic mutations made our patient susceptible to these two disorders, their risk factors, or a certain shared immune disfunction cascade. Secondly neuroinflammatory nature of MS may trigger immune dysregulation and predispose patient for such tumors.^9^ Also malfunctioning of astrocytes cells which is common in both conditions, should be considered. These neuroimmune cells play various function in MS pathophysiology such as causing blood brain barrier disruption with MMP9 upregulation, glial scar formation and microglia regulation.^9^ Further investigation on astrocyte cell's role in inflammation cascade could lead to a better understanding of this condition pathophysiology.

As currently there is no guideline in approaching pediatric MS patients with benign CNS tumors, our case could help physicians in this rare conditions diagnostic and management challenges. When diagnosing such cases, although rare, it is important to consider co‐occurrence of other conditions with overlapping symptoms in differential diagnosis. For instance in our case though MS was mentioned among differential diagnosis, the patient symptoms were attributed to DIA and patient left undiagnosed for MS until her later attack. Another point to consider is to differentiate between rare presentations of MS which could have overlapping presentations to tumors and how to differentiate them. To exemplify it is important to consider overlapping MRI presentation of tumofactive MS MRI lesions and DIA/DIG in similar patient's management. These lesions could also have a mega cystic appearance on MRI which underline the need for other imaging modalities such as Magnetic resonance spectroscopy to avoid unnecessary surgery. It is important for clinicians to perform a through workup in case of doubting tumofactive lesion in order it to not to be confused with tumors.

In terms of treatment challenges, it is important to make a rapid and through decision and consider all possible scenarios. In our patient even though the initial imaging and symptoms are in favor of MS, patient didn't receive treatment until her later attack. Even though this conservative approach may be acceptable for MS treatment, the subsequent course of events in our situation suggests probably earlier treatment strategy could result in better outcome. This highlights the importance of making a thorough examination and choose a tailored patient management approach in co‐occurrence of two distinct complex neurological disorders.

To conclude our case, a non‐typical presentation of DIG/DIA and MS, emphases the necessity for comprehensive evaluation and imaging, earlier treatment, monitoring and follow up for optimize patient outcome.

## AUTHOR CONTRIBUTIONS

**Shima Jahani:** Formal analysis; investigation; methodology; software; visualization; writing – review and editing. **Abdorreza Naser Moghadasi:** Conceptualization; data curation; investigation; supervision; validation; writing – review and editing.

## FUNDING INFORMATION

The authors received no funding for this study.

## CONFLICT OF INTEREST STATEMENT

Authors have declared that no competing interest exists.

## ETHICS STATEMENT

The current study did not require ethical approval in accordance with local ethical guidelines. The study was conducted in accordance with the Helsinki Declaration, and informed consent was obtained from patient and her parents to discuss or publish the details of their disease, their images, and their course of treatment.

## CONSENT

Written informed consent was obtained from the patient to publish this report in accordance with the journal's patient consent policy.

## Data Availability

The data used to support the findings of this study are included within the article.
